# The J-shaped association between the ratio of neutrophil counts to prognostic nutritional index and mortality in ICU patients with sepsis: a retrospective study based on the MIMIC database

**DOI:** 10.3389/fcimb.2025.1603104

**Published:** 2025-07-22

**Authors:** Jiaqi Lou, Hong Kong, Ziyi Xiang, Xiaoyu Zhu, Shengyong Cui, Jiliang Li, Guoying Jin, Neng Huang, Xin Le, Youfen Fan, Sida Xu

**Affiliations:** ^1^ Burn Department, Ningbo No. 2 Hospital, Ningbo, Zhejiang, China; ^2^ Ningbo College of Health Science, Ningbo, Zhejiang, China; ^3^ Institute of Pathology, Faculty of Medicine, University of Bonn, Bonn, Germany; ^4^ Health Science Center, Ningbo University, Ningbo, Zhejiang, China

**Keywords:** intensive care unit, MIMIC-IV database, mortality, sepsis, neutrophil, prognostic nutritional index

## Abstract

**Background:**

The ICU faces persistent challenges with sepsis, marked by systemic inflammation and metabolic disruptions, often leading to poor outcomes. Despite advances, reliable biomarkers for predicting sepsis outcomes are needed. This study introduces a novel indicator combining neutrophil count and prognostic nutritional index (PNI) to improve predictive accuracy by addressing both inflammatory and nutritional-immune aspects.

**Methods:**

We conducted a retrospective cohort study analyzing data from the MIMIC-IV database, focusing on adults diagnosed with sepsis per Sepsis 3.0 criteria. We excluded those younger than 18, with ICU stays under 48 hours, multiple ICU admissions, or incomplete data. Participants’ neutrophil counts/PNI ratios were calculated and correlated with 30, 60, and 90-day hospital and ICU mortality, utilising Kaplan-Meier survival analysis, Cox proportional hazards models, restricted cubic spline (RCS) models and subgroup analysis.

**Results:**

We included 2,116 patients from 22,517 eligible cases. Survival analysis demonstrated lower survival probabilities for higher neutrophil counts/PNI ratios across all observed time windows. Cox regression models revealed a significant association between higher neutrophil counts/PNI ratios and increased short- to medium-term mortality. The restricted cubic spline regression models illustrated a J-shaped relationship between neutrophil counts/PNI and mortality.

**Conclusion:**

The neutrophil counts/PNI ratio is a promising prognostic biomarker for sepsis-related outcomes in ICU settings, offering improved risk stratification and potentially guiding therapeutic interventions. Further research is warranted to validate these findings across diverse populations.

## 1 Background

The intensive care unit (ICU) is a dynamic environment where critically ill patients are managed with the utmost precision due to the complex nature of their conditions. Among these conditions, sepsis remains a pervasive challenge, marked by systemic inflammation and subsequent metabolic disorders. In septic patients, the immune response is heightened, leading to altered physiological states characterized by elevated neutrophil counts and disrupted protein metabolism, manifesting as hypoalbuminemia and lymphopenia (Zhang et al., 2024). These changes contribute to a cascade that exacerbates patient vulnerability, often resulting in poor prognoses. Despite advances in sepsis management, the prognosis remains grim, highlighting a glaring need for reliable biomarkers that can predict outcomes and guide therapeutic strategies. However, the identification and adoption of such biomarkers in clinical settings have been inconsistent, largely due to variability in the disease presentation and complexity of metabolic interactions within these patients (Prucha and Zahorec, 2024).

Recognizing the intricacies of sepsis-related metabolic disorders, our study proposes a novel indicator that harnesses the ratio of neutrophil counts (a marker of acute inflammatory response) to the prognostic nutritional index (PNI, a comprehensive measure integrating albumin levels and lymphocyte counts to reflect nutritional and immunological status). Historically, neutrophil counts have been extensively studied as indicators of inflammation and immune activation, providing insights into the body’s immediate response to infection (Saxena et al., 2024). However, their specificity is limited by confounding factors related to infection severity and comorbidities. Similarly, the PNI has been employed as a prognostic tool in the context of cancer (Zheng et al., 2024) and chronic illnesses (Santos et al., 2025), due to its ability to delineate the nutritional status and immune competence of patients.

Recent studies have also highlighted its potential in acute settings like sepsis, where immediate physiological changes supersede long-term health evaluations. For instance, Toscano et al (Toscano et al., 2025). demonstrated the utility of PNI in predicting clinical outcomes in septic patients, underscoring the importance of integrating nutritional assessment into sepsis management In their study, Toscano et al. evaluated various nutritional scores, including the modified Glasgow Prognostic Score (mGPS), PNI, Controlling Nutritional Status (CONUT) score, modified Nutrition Risk in Critically Ill (mNUTRIC) score, and blood urea nitrogen-to-albumin ratio (BAR), in forecasting mortality and clinical outcomes in patients with sepsis. Among these, the mNUTRIC score emerged as the strongest independent predictor of in-hospital mortality. However, the PNI also showed significant potential in assessing the nutritional and immune status of septic patients, which aligns with our study’s focus on combining neutrophil counts with PNI to enhance predictive accuracy.

By combining these two parameters, we aim to overcome their individual limitations and enhance predictive accuracy in septic patients. The logic of this combination lies in addressing both the inflammatory state (via neutrophil counts) and the nutritional-immune interplay (captured by the PNI). This dual approach enables a more nuanced perspective on patient conditions, offering insights not only into their current inflammatory state but also into underlying nutritional and immune support. The expected benefits of this composite indicator include improved risk stratification, more tailored treatment regimens, and ultimately, enhanced patient outcomes. With this fusion, we seek to bridge existing gaps in biomarker utility, offering a more coherent and clinically relevant tool for ICU practitioners.

Our research draws upon data from the Medical Information Mart for Intensive Care (MIMIC) database, a comprehensive collection of de-identified health data, including demographic, laboratory, and clinical information from thousands of ICU patients (Ning et al., 2024). The MIMIC database is noted for its robustness and diversity, representing an invaluable resource for retrospective analyses concerning critical care and beyond. Its extensive dataset allows for rigorous validation and generalizability of research findings across varying hospital settings and patient populations. This backdrop affords us a unique opportunity to evaluate our new indicator within a richly detailed cohort of septic patients in the ICU context. The primary objectives of our study are twofold: first, to ascertain the predictive utility of the neutrophil-to-PNI ratio regarding sepsis outcomes, and second, to establish this novel indicator as a practical and reliable tool in routine ICU assessments. We specifically focused on sepsis as an exemplar condition for investigating the neutrophil-to-PNI ratio due to its distinctive pathophysiological duality. Sepsis triggers a self-amplifying cycle of dysregulated inflammation and catastrophic metabolic collapse, uniquely disrupting core biological domains reflected by this composite biomarker: Sepsis induces profound neutrophil dysfunction—characterized by impaired chemotaxis and delayed apoptosis (Saxena et al., 2024; Santos et al., 2025)—that paradoxically fuels organ injury while failing to clear pathogens. Concurrently, sepsis-driven hypercatabolism (Saxena et al., 2024) rapidly depletes visceral proteins, crippling adaptive immunity and tissue repair capacity. This synergistic pathophysiology creates a critical context where the neutrophil-to-PNI ratio transcends being a mere prognostic indicator. By quantifying the balance between maladaptive inflammation (numerator) and nutritional-immune competence (denominator), the ratio captures the core biological tension defining sepsis severity. While other critical illnesses may alter individual components (e.g., isolated neutrophilia in trauma or hypoalbuminemia in cirrhosis), sepsis’s unique confluence of immune paralysis and metabolic failure establishes this composite biomarker as a mechanistically grounded tool for risk stratification and potential therapeutic targeting—offering insights unlikely to be replicated in conditions lacking this pathophysiological nexus. By validating our indicator through a systematic examination of MIMIC data, we aim to deliver insights that could redefine prognostic evaluations for septic patients. The anticipated results of our study promise to have far-reaching implications in clinical practice, potentially streamlining decision-making processes, optimizing resource allocation, and enhancing patient-tailored approaches to sepsis management.

## 2 Methods

### 2.1 Data source and study design

We carried out a retrospective cohort study using data from MIMIC IV (version 2.2). This database integrates two key systems: a comprehensive hospital-wide electronic health record (EHR) and an ICU-specific clinical information system, encompassing data from 2008 to 2024 (Ning et al., 2024). Access to the database was authorized for one of the authors (JQ L), who obtained the required authentication and successfully completed the Collaborative Institutional Training Initiative examination (authentication number 60691748). For our study, we extracted relevant variables, and patient data were de-identified to protect privacy. Given the retrospective design and anonymized nature of the patient information within the database, the Human Research Ethics Committee of Ningbo No. 2 Hospital waived the necessity for informed consent.

Due to the retrospective nature of the study, we did not perform traditional sample size calculations or power analyses. Nonetheless, the abundance of records available in the database was considered sufficient to fulfill the study’s aims. This conclusion is informed by our examination of the relationship between the neutrophil count to prognostic nutritional index ratio and mortality outcomes within a large, diverse patient cohort. The convenience samples drawn from the database adequately represent the intensive care population, thereby validating the statistical analyses conducted in this study (He and Qiu, 2024).

### 2.2 Participants

**Figure 1:**
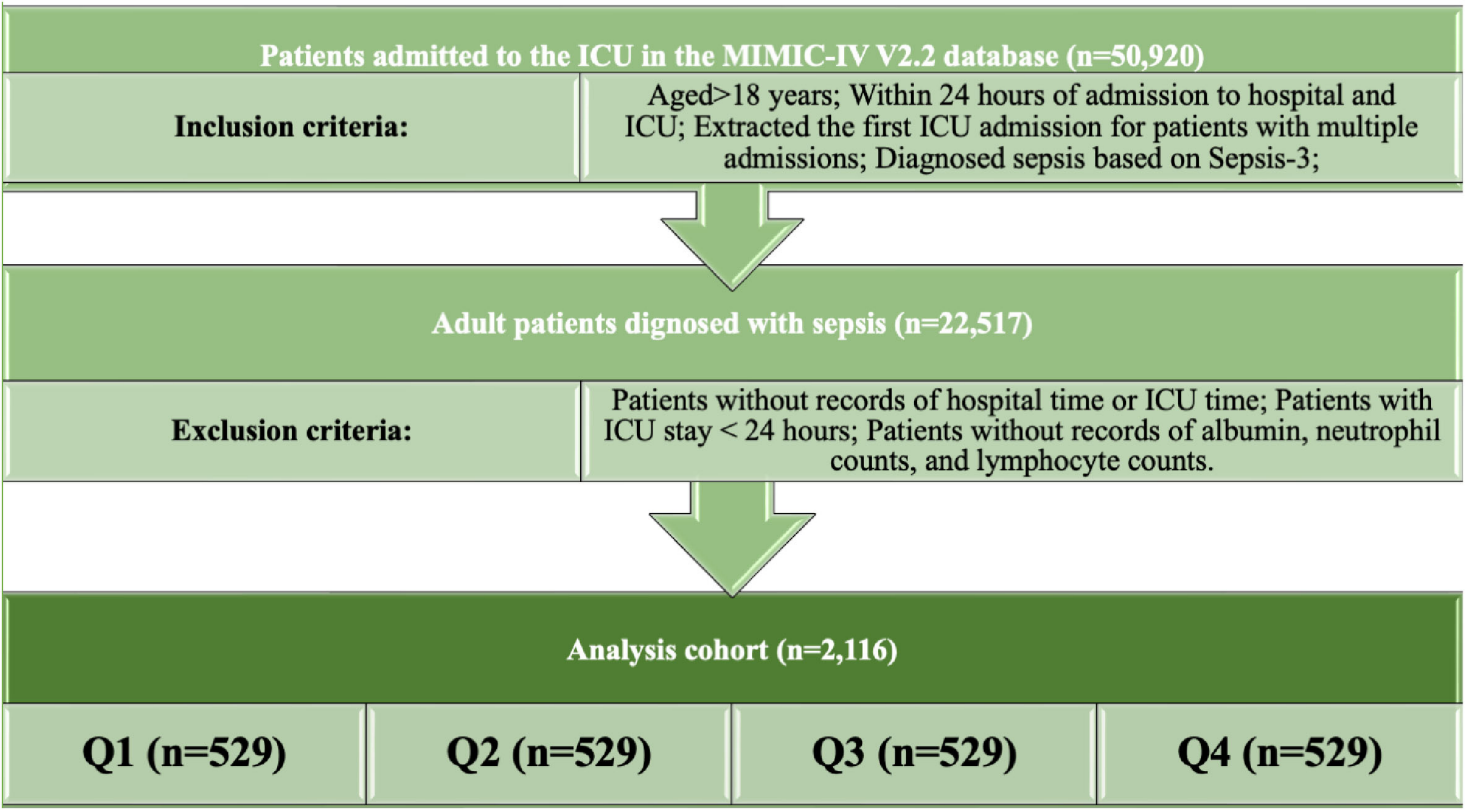
Selection of the population from the MIMIC-IV database in this study.

The study included all sepsis patients from the MIMIC IV version 2.2 database. Sepsis was defined according to the Sepsis 3.0 criteria (Wang et al., 2023), as set by the American Society for Critical Care Medicine (SCCM) and the European Society for Critical Care Medicine (ESICM). We extracted patient data using PostgreSQL (Feng et al., 2025), selecting those who met the inclusion criteria: sepsis patients over the age of 18 who were admitted to the ICU for the first time. The exclusion criteria were as follows: (1) patients younger than 18 years; (2) ICU stays of less than 48 hours; (3) patients with multiple ICU admissions due to sepsis; and (4) cases with incomplete data, specifically missing records for albumin, neutrophil counts, and lymphocyte counts (**Figure 1**).

### 2.3 Research procedures and definitions

Data extraction from the MIMIC-IV database was conducted using Structured Query Language (SQL) via Navicat Premium. The extraction focused on patient demographics, medical history, and initial laboratory test results. Below are the whole parameters extracted from database:

Demographic and basic clinical characteristics included age (Mean ± SD), weight (Mean ± SD), height (Mean ± SD), gender (n (%)), race (n (%)), insurance (n (%)), language (n (%)), and marital status (n (%)).

Laboratory parameters comprised white blood cell count (WBC) (×10^9^/L), red blood cell count (RBC) (×10^2^/L), red blood cell distribution width (RDW) (%), albumin (g/L), sodium (mmol/L), potassium (mmol/L), total calcium (mmol/L), chlorine (mmol/L), glucose (mmol/L), blood pH, thrombin time (TT) (s), prothrombin time (PT) (s), partial thromboplastin time (PTT) (s), international normalized ratio (INR), alanine aminotransferase (ALT) (U/L), aspartate aminotransferase (AST) (U/L), platelet count (×10^9^/L), hemoglobin (g/dL), hematocrit (HCT) (%), globulin (g/dL), glycated hemoglobin A1c (HbA1c) (%), triglyceride (mg/dL), total cholesterol (mg/dL), high-density lipoprotein (HDL) (mg/dL), and low-density lipoprotein (LDL) (mg/dL).

Treatment and intervention indicators included continuous renal replacement therapy (CRRT) (n (%)), mechanical ventilation (n (%)), duration of continuous renal replacement therapy (days), and duration of mechanical ventilation (hours).

Comorbidity and disease history covered hypertension (HTN) (n (%)), type 2 diabetes mellitus (T2DM) (n (%)), heart failure (HF) (n (%)), myocardial infarction (MI) (n (%)), malignant tumor (n (%)), chronic kidney disease (CKD) (n (%)), acute renal failure (ARF) (n (%)), cirrhosis (n (%)), hepatitis (n (%)), pneumonia (n (%)), stroke (n (%)), hyperlipemia (n (%)), chronic obstructive pulmonary disease (COPD) (n (%)), and acute kidney injury (AKI) (n (%)).

Severity scores included the Sequential Organ Failure Assessment Score (SOFA), Acute Physiology and Chronic Health Evaluation III Score (APACHE III), Simplified Acute Physiology Score II (SAPS II), Oasis Score (OASIS), and Glasgow Coma Scale Score (GCS).

These parameters comprehensively cover patient characteristics, laboratory test results, treatment measures, comorbidity status, and disease severity scores.

During the data cleaning process, any predictors with more than 30% missing data were excluded from the analysis. The Neutrophil counts/Prognostic Nutritional Index was determined using the formula: Neutrophil counts/Prognostic Nutritional Index = Neutrophil counts (×10^9^/L)/[serum albumin (g/dL) + 5 × lymphocyte counts (×10^9^/L)] (Chen et al., 2025).

### 2.4 Outcomes and measures

In this study, we aimed to evaluate the predictive capacity of this measure for short- to medium-term mortality in patients, with a primary focus on hospital and ICU mortality at 30, 60, and 90 days.

### 2.5 Statistical analysis

Continuous variables were represented as mean ± standard deviation for normally distributed data, or as median with interquartile range for non-normally distributed data. Categorical variables were reported as frequency and percentage. For normally distributed data, t-tests or analysis of variance (ANOVA) were applied to analyze differences (Fang et al., 2025). In contrast, data not following a normal distribution were examined using the Mann-Whitney U test or the Kruskal-Wallis test. Kaplan-Meier survival analysis was employed to evaluate the incidence of endpoint events across different levels of the Neutrophil counts/Prognostic Nutritional Index, with differences assessed using the log-rank test. Kaplan-Meier curves offer a visual representation of survival differences among groups or conditions (Wang et al., 2025b), and their application is flexible as they do not require assumptions about the data distribution.

We employed the Cox proportional hazards model to calculate hazard ratios (HR) and 95% confidence intervals (CI) for the association between Neutrophil counts/PNI and endpoint outcomes. This model is well-suited for evaluating multiple factors influencing survival, and it accommodates censored survival data without requiring specific assumptions about survival distribution types (Wang et al., 2025a). The Neutrophil counts/PNI was examined both as a continuous variable and divided into quartiles. Our analysis involved three models: Model 1 involved univariate analysis; Model 2 was adjusted for basic demographic variables including age, gender, height, weight, race, language, insurance status, and marital status; and Model 3 included further adjustments for clinical and laboratory parameters such as white blood cell count, red blood cell count, platelet count, hemoglobin, chloride, and critical clinical interventions like continuous renal replacement therapy and mechanical ventilation. Additionally, it accounted for comorbid conditions such as hypertension, type 2 diabetes mellitus, heart failure, myocardial infarction, malignant tumors, chronic kidney disease, acute renal failure, cirrhosis, hepatitis, pneumonia, stroke, hyperlipidemia, acute kidney injury, and chronic obstructive pulmonary disease. Severity scores such as the SOFA score, APSIII score, SAPSII score, OASIS score, and Charlson score were also included.

To explore the nonlinear relationship between baseline Neutrophil counts/PNI and mortality in both hospital and ICU settings, we applied the restricted cubic spline (RCS) regression model (Chen et al., 2025). Neutrophil counts/PNI was treated as either a continuous or an ordered variable, with the first quartile serving as the reference group. P-values for trends across quartiles were calculated. RCS is a non-parametric and flexible technique that models survival curves by transforming survival times into piecewise functions at specific nodes, which allows for the modeling of various survival time distributions without imposing rigid assumptions (Chen et al., 2025).

Subgroup analyses were conducted to explore potential differences across various subgroups based on age (≤70 years old and >70 years old), gender, BMI (<27.4 kg/m², 27.4-31.2 kg/m², ≥31.2 kg/m²), presence of hypertension, type 2 diabetes, hepatitis, mechanical ventilation and CRRT. These analyses evaluated the consistency of the Neutrophil counts/PNI’s prognostic value for the primary outcomes. Cox models were also used in subgroup analyses to adjust for all baseline variables.

The Geriatric Nutritional Risk Index (GNRI) is designed specifically for the physiological characteristics and nutritional needs of older adults. GNRI takes into account the patient’s weight and protein nutritional status, and is suitable for various types of elderly patients, including those with unclear consciousness or critical conditions. It can more comprehensively assess the nutritional risks of older adults. In order to better explore promising and more suitable prognostic markers in the elderly population with severe sepsis (>=70 years), we also conducted Cox analysis of the relationship between hospital and ICU mortality rates using Neutrophil counts/Geriatric Nutritional Risk Index (NC/GNRI) for elderly patients with severe sepsis aged 70 years or older. Among them, the GNRI formula is: GNRI=14.89 × Serum albumin (g/dL)+41.7 × (Actual weight/Ideal weight). Ideal weight (kg) =22 × Height (m) × Height (m). If the actual weight is greater than the ideal weight, it will be calculated according to the ideal weight. The setting of the model remains the same as before.

Data processing and analysis were executed using R version 4.3.0, with statistical significance set at P<0.05 for two-tailed tests. Multiple imputation, utilizing the “mice” R package, was employed to address missing values. Features with missing values exceeding 30% were removed prior to imputation. This package implements the multivariate imputation by chained equations method, which is extensively used in data preprocessing (Meng et al., 2024). Compared with simple mean substitution, this advanced imputation approach based on it is more accurate under the assumption that data are missing at random. Models were constructed according to variable types to generate predicted values for replacing missing data. Subsequently, subgroup analyses were conducted using the “jstable” R package, which facilitates multiple subgroup analyses for generalized linear models (Li et al., 2025), Cox regression models, and other models, producing standardized tables and visualizations such as forest plots.

## 3 Results

### 3.1 Characteristics of included patients

Among the adult patients in the MIMIC-IV database, a total of 22,517 subjects met the eligibility criteria. Initially, 192 potential prognostic factors were identified from the database. After data cleaning, we excluded 135 predictors that had more than 30% missing data. Consequently, 57 prognostic factors were retained for inclusion in the final model for analysis. After a standardized screening process, 2,116 patients with sepsis who had complete data for subsequent analyses were included. The mean (interquartile range) of Neutrophil counts/PNI was 1.70 (1.38-2.34). Subsequently, Neutrophil counts/PNI were divided into four quartiles, labeled Q1 (Quartile 1; Neutrophil counts/PNI ≤ 0.72, n=529), Q2 (Quartile 2; 0.72 < Neutrophil counts/PNI ≤ 1.28, n=529), Q3 (Quartile 3; 1.28 < Neutrophil counts/PNI ≤ 2.10, n=529) and Q4 (Quartile 4; Neutrophil counts/PNI > 2.10, n=529). The screening procedure is illustrated in **Figure 1**, and the baseline characteristics of all enrolled patients are summarized in **Table 1**. Comparisons of baseline data among the four groups revealed significant differences in body weight, race, white blood cell count, red blood cell distribution width, platelet count, albumin level, hemoglobin concentration, sodium, potassium, total calcium, chloride, glucose, pH, thrombin time, prothrombin time, international normalized ratio, alanine aminotransferase, aspartate aminotransferase, SOFA score, APACHE III score, SAPS II score, OASIS score, hypertension prevalence, heart failure incidence, myocardial infarction occurrence, and chronic kidney disease presence (P < 0.05).

**Table 1:** Crucial characteristics and outcomes of participants categorized by the Neutrophil counts/PNI.

Variables	Total (n=2116)	Q1 (n=529)	Q2 (n=529)	Q3 (n=529)	Q4 (n=529)		Statistic			*P*
Characteristics										
Age, Mean ± SD	65.05 ± 16.78	64.28 ± 16.43	64.21 ± 17.38	65.41 ± 16.55	66.31 ± 16.69		F=1.88			0.131
Weight, Mean ± SD	82.70 ± 25.28	81.50 ± 23.15	80.52 ± 24.34	85.91 ± 27.76	82.84 ± 25.38		F=4.54			0.004
Height, Mean ± SD	169.56 ± 10.41	170.25 ± 11.33	168.36 ± 10.07	169.65 ± 10.30	170.05 ± 9.91		F=2.22			0.084
Gender, n (%)							χ²=5.33			0.149
F	909 (42.96)	232 (43.86)	246 (46.50)	220 (41.59)	211 (39.89)					
M	1207 (57.04)	297 (56.14)	283 (53.50)	309 (58.41)	318 (60.11)					
Race, n (%)							χ²=48.65			**<0.001**
American	9 (0.43)	2 (0.38)	3 (0.57)	3 (0.57)	1 (0.19)					
Asian	73 (3.45)	21 (3.97)	14 (2.65)	11 (2.08)	27 (5.10)					
Black	178 (8.41)	69 (13.04)	29 (5.48)	39 (7.37)	41 (7.75)					
Hispanic/Latino	54 (2.55)	17 (3.21)	15 (2.84)	10 (1.89)	12 (2.27)					
Other	83 (3.92)	16 (3.02)	30 (5.67)	14 (2.65)	23 (4.35)					
Insurance, n (%)							χ²=4.82			0.567
Medicaid	151 (7.14)	44 (8.32)	39 (7.37)	36 (6.81)	32 (6.05)					
Medicare	915 (43.24)	228 (43.10)	213 (40.26)	235 (44.42)	239 (45.18)					
Other	1050 (49.62)	257 (48.58)	277 (52.36)	258 (48.77)	258 (48.77)					
Language, n (%)							χ²=2.64			0.450
Other	212 (10.02)	60 (11.34)	48 (9.07)	47 (8.88)	57 (10.78)					
English	1904 (89.98)	469 (88.66)	481 (90.93)	482 (91.12)	472 (89.22)					
Marital Status, n (%)							χ²=16.32			0.177
Divorced	148 (6.99)	44 (8.32)	40 (7.56)	34 (6.43)	30 (5.67)					
Married	782 (36.96)	217 (41.02)	177 (33.46)	202 (38.19)	186 (35.16)					
Unknown	392 (18.53)	80 (15.12)	105 (19.85)	97 (18.34)	110 (20.79)					
Single	575 (27.17)	143 (27.03)	145 (27.41)	143 (27.03)	144 (27.22)					
Widowed	219 (10.35)	45 (8.51)	62 (11.72)	53 (10.02)	59 (11.15)					
Laboratory parameters										
WBC (×10^9^/L)	14.66 ± 11.21	9.80 ± 14.48	12.02 ± 6.93	15.11 ± 6.21	21.73 ± 11.21		F=135.12			**<0.001**
RBC (×10^12^/L)	3.40 ± 0.75	3.33 ± 0.77	3.43 ± 0.76	3.42 ± 0.75	3.40 ± 0.73		F=1.67			0.171
RDW (%)	15.93 ± 2.85	16.05 ± 3.11	15.57 ± 2.73	15.79 ± 2.64	16.30 ± 2.84		F=6.60			**<0.001**
Albumin (g/L)	2.99 ± 0.65	3.11 ± 0.62	3.13 ± 0.66	2.96 ± 0.60	2.75 ± 0.63		F=40.98			**<0.001**
Sodium (mmol/L)	138.59 ± 5.49	139.16 ± 5.37	139.03 ± 5.35	138.12 ± 5.78	138.03 ± 5.37		F=6.17			**<0.001**
Potassium (mmol/L)	4.26 ± 0.64	4.17 ± 0.58	4.24 ± 0.61	4.29 ± 0.66	4.35 ± 0.68		F=8.25			**<0.001**
Total calcium (mmol/L)	8.20 ± 0.82	8.24 ± 0.80	8.32 ± 0.83	8.18 ± 0.78	8.07 ± 0.83		F=9.16			**<0.001**
Chlorine (mmol/L)	102.83 ± 6.49	103.45 ± 6.36	103.14 ± 6.53	102.35 ± 6.59	102.38 ± 6.44		F=3.83			**0.010**
Glucose (mmol/L)	150.44 ± 64.63	143.73 ± 67.25	145.02 ± 54.17	158.18 ± 71.67	154.82 ± 63.12		F=6.53			**<0.001**
Ph	7.35 ± 0.08	7.36 ± 0.09	7.36 ± 0.07	7.35 ± 0.08	7.34 ± 0.08		F=8.72			**<0.001**
TT (S)	46.18 ± 58.89	NaN ± NA	17.30 ± 2.23	49.70 ± 66.90	82.45 ± 95.53		F=0.69			0.538
PT (s)	17.78 ± 10.11	17.00 ± 10.38	17.09 ± 9.49	17.97 ± 8.97	19.06 ± 11.35		F=4.72			**0.003**
PTT (s)	39.65 ± 19.06	37.85 ± 16.76	39.68 ± 19.90	39.48 ± 18.49	41.60 ± 20.74		F=3.35			**0.018**
INR	1.62 ± 0.88	1.54 ± 0.79	1.57 ± 0.86	1.65 ± 0.81	1.74 ± 1.01		F=5.98			**<0.001**
ALT (U/L)	197.87 ± 762.64	181.36 ± 675.56	159.06 ± 471.44	218.99 ± 1022.97	232.20 ± 778.20		F=1.01			0.387
AST (U/L)	358.55 ± 1361.71	280.24 ± 1019.17	315.69 ± 1028.18	410.60 ± 1748.81	427.92 ± 1503.69		F=1.46			0.224
Platelet (×10^9^/L)	178.66 ± 106.19	147.05 ± 95.89	180.09 ± 99.20	189.54 ± 99.90	197.97 ± 121.07		F=24.10			**<0.001**
Hemoglobin (g/dL)	10.08 ± 2.13	9.92 ± 2.08	10.24 ± 2.14	10.17 ± 2.18	10.02 ± 2.09		F=2.47			0.060
Hematocrit (%)	31.05 ± 6.44	30.58 ± 6.55	31.43 ± 6.40	31.26 ± 6.50	30.94 ± 6.29		F=1.80			0.145
Globulin (g/dL)	2.71 ± 1.13	2.98 ± 1.66	2.54 ± 0.88	2.65 ± 0.58	2.54 ± 0.72		F=0.99			0.402
Glycated Hemoglobin A1c (%)	6.35 ± 1.78	6.35 ± 1.61	6.54 ± 1.98	6.25 ± 1.74	6.16 ± 1.79		F=0.61			0.609
Triglyceride (mg/dL)	209.68 ± 356.91	226.73 ± 563.75	222.42 ± 328.48	172.50 ± 147.63	217.75 ± 188.47		F=0.54			0.655
Total cholesterol (mg/dL)	143.96 ± 58.21	147.41 ± 59.19	147.50 ± 56.11	145.10 ± 60.65	125.70 ± 54.38		F=1.08			0.358
High-density lipoprotein (mg/dL)	43.72 ± 20.84	42.41 ± 19.74	45.69 ± 19.18	43.77 ± 24.07	43.20 ± 21.02		F=0.25			0.861
Low-density lipoprotein (mg/dL)	78.73 ± 46.67	80.89 ± 47.11	83.08 ± 46.78	77.53 ± 50.21	65.57 ± 36.19		F=0.81			0.488
Treatment										
CRRT (n (%))							χ²=7.83			0.050
Yes	227 (10.73)	46 (8.70)	49 (9.26)	61 (11.53)	71 (13.42)					
No	1889 (89.27)	483 (91.30)	480 (90.74)	468 (88.47)	458 (86.58)					
Ventilation (n (%))							χ²=4.40			0.221
Yes	1750 (82.70)	427 (80.72)	445 (84.12)	448 (84.69)	430 (81.29)					
No	366 (17.30)	102 (19.28)	84 (15.88)	81 (15.31)	99 (18.71)					
CRRT (days)	6.16 ± 5.84	5.04 ± 4.59	5.98 ± 5.84	6.15 ± 5.51	7.03 ± 6.75		F=1.10			0.350
Ventilation (hours)	87.97 ± 107.38	80.96 ± 103.82	85.07 ± 93.01	99.67 ± 117.07	85.75 ± 113.42		F=2.56			0.054
Comorbidity										
Hypertension (n (%))							χ²=7.89			**0.048**
No	1397 (66.02)	323 (61.06)	358 (67.67)	361 (68.24)	355 (67.11)					
Yes	719 (33.98)	206 (38.94)	171 (32.33)	168 (31.76)	174 (32.89)					
Type 2 diabetes mellitus (n (%))							χ²=2.79			0.425
No	1526 (72.12)	389 (73.53)	368 (69.57)	380 (71.83)	389 (73.53)					
Yes	590 (27.88)	140 (26.47)	161 (30.43)	149 (28.17)	140 (26.47)					
Heart failure (n (%))							χ²=12.44			**0.006**
No	1462 (69.09)	396 (74.86)	347 (65.60)	354 (66.92)	365 (69.00)					
Yes	654 (30.91)	133 (25.14)	182 (34.40)	175 (33.08)	164 (31.00)					
Myocardial Infarction (n (%))							χ²=8.42			**0.038**
No	1753 (82.84)	458 (86.58)	424 (80.15)	432 (81.66)	439 (82.99)					
Yes	363 (17.16)	71 (13.42)	105 (19.85)	97 (18.34)	90 (17.01)					
Malignant Tumor (n (%))							χ²=15.68			**0.001**
No	1790 (84.59)	456 (86.20)	451 (85.26)	463 (87.52)	420 (79.40)					
Yes	326 (15.41)	73 (13.80)	78 (14.74)	66 (12.48)	109 (20.60)					
Chronic kidney disease (n (%))							χ²=9.83			**0.020**
No	1649 (77.93)	438 (82.80)	401 (75.80)	405 (76.56)	405 (76.56)					
Yes	467 (22.07)	91 (17.20)	128 (24.20)	124 (23.44)	124 (23.44)					
Acute renal failure (n (%))							χ²=54.31			**<0.001**
No	887 (41.92)	273 (51.61)	249 (47.07)	199 (37.62)	166 (31.38)					
Yes	1229 (58.08)	256 (48.39)	280 (52.93)	330 (62.38)	363 (68.62)					
Cirrhosis (n (%))							χ²=9.80			**0.020**
No	1772 (83.74)	421 (79.58)	456 (86.20)	445 (84.12)	450 (85.07)					
Yes	344 (16.26)	108 (20.42)	73 (13.80)	84 (15.88)	79 (14.93)					
Hepatitis (n (%))							χ²=4.04			0.258
No	1863 (88.04)	462 (87.33)	476 (89.98)	456 (86.20)	469 (88.66)					
Yes	253 (11.96)	67 (12.67)	53 (10.02)	73 (13.80)	60 (11.34)					
Pneumonia (n (%))							χ²=8.92			**0.030**
No	1250 (59.07)	340 (64.27)	309 (58.41)	294 (55.58)	307 (58.03)					
Yes	866 (40.93)	189 (35.73)	220 (41.59)	235 (44.42)	222 (41.97)					
Stroke (n (%))							χ²=1.93			0.587
No	1991 (94.09)	504 (95.27)	496 (93.76)	497 (93.95)	494 (93.38)					
Yes	125 (5.91)	25 (4.73)	33 (6.24)	32 (6.05)	35 (6.62)					
Hyperlipemia (n (%))							χ²=3.75			0.290
No	1432 (67.67)	375 (70.89)	349 (65.97)	350 (66.16)	358 (67.67)					
Yes	684 (32.33)	154 (29.11)	180 (34.03)	179 (33.84)	171 (32.33)					
COPD (n (%))							χ²=6.20			0.102
No	1814 (85.73)	470 (88.85)	453 (85.63)	446 (84.31)	445 (84.12)					
Yes	302 (14.27)	59 (11.15)	76 (14.37)	83 (15.69)	84 (15.88)					
Acute kidney injury (n (%))							χ²=4.53			0.210
No	414 (19.57)	111 (20.98)	110 (20.79)	87 (16.45)	106 (20.04)					
Yes	1702 (80.43)	418 (79.02)	419 (79.21)	442 (83.55)	423 (79.96)					
*Scoring systems*										
SOFA score (score)	7.63 ± 4.07	7.17 ± 3.95	7.25 ± 3.94	7.75 ± 4.15	8.36 ± 4.14		F=9.84			**<0.001**
APSIII score (score)	57.09 ± 23.34	52.91 ± 23.29	52.93 ± 22.16	59.22 ± 23.30	63.30 ± 22.96		F=26.13			**<0.001**
SAPSII score (score)	43.19 ± 15.25	40.73 ± 14.94	40.88 ± 14.20	43.95 ± 15.21	47.19 ± 15.72		F=21.81			**<0.001**
OASIS score (score)	35.45 ± 8.83	33.94 ± 8.83	34.98 ± 8.32	36.14 ± 8.81	36.74 ± 9.09		F=10.65			**<0.001**
GCS score (score)	13.47 ± 2.85	13.53 ± 2.67	13.41 ± 2.93	13.33 ± 3.05	13.60 ± 2.72		F=0.95			0.415

The Neutrophil counts/PNI ratio was divided into quartiles as follows: Q1 (Quartile 1; Neutrophil counts/PNI ≤ 0.72, n=529), Q2 (Quartile 2; 0.72 < Neutrophil counts/PNI ≤ 1.28, n=529), Q3 (Quartile 3; 1.28 < Neutrophil counts/PNI ≤ 2.10, n=529), Q4 (Quartile 4; Neutrophil counts/PNI > 2.10, n=529). Continuous variables are expressed as the median and interquartile range. Counting data are presented as numbers and percentages. The medical condition was defined based on the ICD-9 code. F: ANOVA, χ²: Chi-square test; SD, standard deviation; WBC, white blood cell; RBC, red blood cell; RDW, red blood cell distribution width; CRRT, continuous renal replacement therapy; TT, ThrombinTime; PT, ProthrombinTime; PTT, partial thromboplastin time; INR, International Normalized Ratio; ALT, Alanine aminotransferase; AST, Aspartate aminotransferase; COPD, Chronic obstructive pulmonary disease.

Bold red font indicates P<0.05, indicating statistical significance.

### 3.2 Kaplan‑Meier survival curve analysis

**Figure 2:**
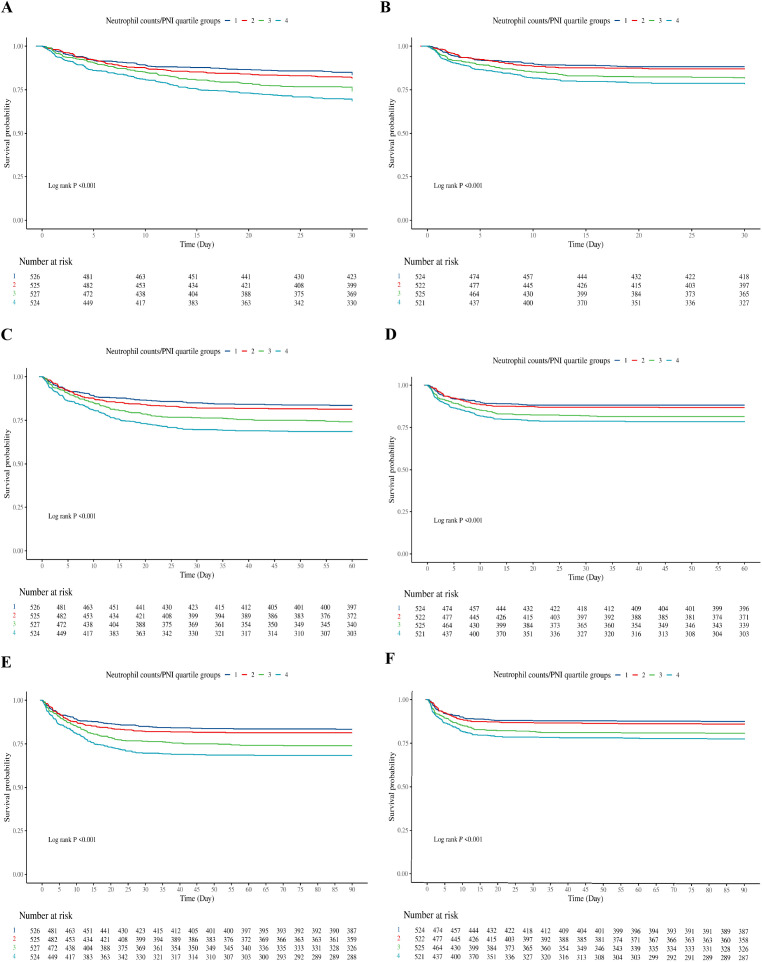
Kaplan-Meier survival curve of cumulative survival rate during hospitalization and ICU for groups. **(A)**: Kaplan-Meier survival curve of cumulative survival rate during hospitalization for groups at 30 days. **(B)**: Kaplan-Meier survival curve of cumulative survival rate during ICU for groups at 30 days. **(C)**: Kaplan-Meier survival curve of cumulative survival rate during hospitalization for groups at 60 days. **(D)**: Kaplan-Meier survival curve of cumulative survival rate during ICU for groups at 60 days. **(E)**: Kaplan-Meier survival curve of cumulative survival rate during hospitalization for groups at 90 days. **(F)**: Kaplan-Meier survival curve of cumulative survival rate during ICU for groups at 90 days.

The Kaplan-Meier survival curves, as depicted in **Figure 2**, demonstrate a steadily declining survival probability for critically ill sepsis patients from the first quartile (Q1) to the fourth quartile (Q4) in both ICU and hospital settings. Significant differences in mortality among the quartile groups were observed during ICU admission and across the hospitalization period, with log-rank test results showing P<0.001 for both comparisons. These differences are evident at 30 days (**Figures 2A, B**), 60 days (**Figures 2C, D**), and 90 days (**Figures 2E, F**).

### 3.3 Cox regression models for all-cause mortality (in hospital and ICU)

In the Cox regression analysis, when Neutrophil counts/PNI was added as a continuous variable to the univariate regression analysis, We found that in Model 1(HR 1.06 [95%CI 1.04 to 1.09], P<0.001), Model 2(HR 1.04 [95%CI 1.01 to 1.07], P=0.035) and Model 3(HR 1.04 [95% CI 1.01 to 1.07], P=0.018) were associated with increased 30-day hospital mortality. In addition, increased Neutrophil counts/PNI in Model 1(HR 1.06 [95% CI 1.03 to 1.08], P<0.001) was also associated with increased 30-day ICU mortality. Secondly, In Model 1(HR 1.06 [95% CI 1.04 to 1.09], P<0.001), Model 2(HR 1.04 [95% CI 1.01 to 1.07], P=0.035) and Model 3(HR 1.04 [95% CI 1.01 to 1.07], P=0.017) were associated with increased 60-day hospital mortality. Increased 30-day ICU mortality in Model 1 was also associated with increased Neutrophil counts/PNI (HR 1.06 [95% CI 1.03 to 1.08], P<0.001). Finally, Model 1 was also observed for 90-day in-hospital mortality (HR 1.06 [95% CI 1.04 to 1.09], P<0.001), Model 2(HR 1.04 [95%CI 1.01 to 1.07], P=0.035) and Model 3(HR 1.04 [95%CI 1.01 to 1.07], P=0.035), P=0.017) were associated with elevated Neutrophil counts/PNI. 90-day ICU mortality was also associated with Neutrophil counts/PNI in Model 1 (HR 1.06 [95% CI 1.03 to 1.08], P<0.001).

When the Neutrophil counts/PNI was segmented into four quartile groups for analysis within the three models, distinct trends were observed in mortality rates:

Model 1: For 30-day in-hospital mortality, the Q3 group had a 63% higher mortality rate compared to Q1 (HR 1.63, 95% CI 1.24-2.14, P<0.001), while the Q4 group had a 108% higher rate than Q1 (HR 2.08, 95% CI 1.60-2.71, P<0.001). Similar patterns were observed at 60 and 90 days, with the Q3 group consistently showing a 63% increase and the Q4 group a 108% rise in mortality compared to Q1 (HR values and CIs consistent for all three time points, P<0.001). Similar trends were observed for ICU mortality, with 30-day mortality 62% higher in Q3 and 95% higher in Q4 compared to Q1 (HR 1.62, 95% CI 1.17-2.23, P=0.003 and HR 1.95, 95% CI 1.43-2.67, P<0.001, respectively). These results were consistent at 60 and 90 days.

Model 2: After adjusting for demographic factors, 30-day in-hospital mortality was 51% higher in Q3 compared to Q1 (HR 1.51, 95% CI 1.15-1.99, P=0.003) and 74% higher in Q4 compared to Q1 (HR 1.73, 95% CI 1.30-2.33, P<0.001). These findings were consistent at 60 and 90 days. Similarly, ICU mortality was 52% higher in Q3 and 68% higher in Q4 compared to Q1 at 30 days (HR 1.52, 95% CI 1.10-2.11, P=0.012 and HR 1.68, 95% CI 1.18-2.37, P=0.004, respectively), with similar trends evident at 60 and 90 days.

Model 3: With further adjustments for clinical and comorbid conditions, 30-day in-hospital mortality in the Q3 group was 72% higher than in the Q1 group (HR 1.72, 95% CI 1.40-2.10, P<0.001), and 67% higher in the Q4 group (HR 1.67, 95% CI 1.38-2.02, P<0.001). Results were comparable at 60 and 90 days. For ICU mortality, the Q3 group exhibited a 33% increase at 30, 60, and 90 days compared to Q1 (HR 1.33, 95% CI 1.05-1.68, P=0.019) (**Table 2**).

**Table 2:** The association between Neutrophil counts/PNI and in-hospital and ICU mortality.

Exposure	Model 1		Model 2		Model 3	
	HR (95% CI)	*P*-value	HR (95% CI)	*P*-value	HR (95% CI)	*P*-value
In-hospital mortality at 30-day						
Neutrophil counts/PNI as continuous	1.06 (1.04 ~ 1.09)	**<0.001**	1.04 (1.01 ~ 1.07)	**0.035**	1.04 (1.01 ~ 1.07)	**0.018**
Q1	1.00 (Reference)		1.00 (Reference)		1.00 (Reference)	
Q2	1.14 (0.85 ~ 1.52)	0.381	1.09 (0.81 ~ 1.46)	0.576	1.03 (0.82 ~ 1.29)	0.791
Q3	1.63 (1.24 ~ 2.14)	**<0.001**	1.51 (1.15 ~ 1.99)	**0.003**	1.72 (1.40 ~ 2.10)	**<0.001**
Q4	2.08 (1.60 ~ 2.71)	**<0.001**	1.74 (1.30 ~ 2.3)	**<0.001**	1.67 (1.38 ~ 2.02)	**<0.001**
ICU mortality at 30-day						
Neutrophil counts/PNI as continuous	1.06 (1.03 ~ 1.08)	**<0.001**	1.03 (0.99 ~ 1.08)	0.122	1.02 (0.99- 1.06)	0.189
Q1	1.00 (Reference)		1.00 (Reference)		1.00 (Reference)	
Q2	1.13 (0.80 ~ 1.59)	0.501	1.09 (0.77 ~ 1.54)	0.633	1.01 (0.77 ~ 1.32)	0.950
Q3	1.62 (1.17 ~ 2.23)	**0.003**	1.52 (1.10 ~ 2.11)	**0.012**	1.33 (1.05 ~ 1.68)	**0.019**
Q4	1.95 (1.43 ~ 2.67)	**<0.001**	1.68 (1.18 ~ 2.37)	**0.014**	1.25 (1.00 ~ 1.57)	0.055
In-hospital mortality at 60-day						
Neutrophil counts/PNI as continuous	1.06 (1.04 ~ 1.09)	**<0.001**	1.04 (1.01 ~ 1.07)	**0.035**	1.04 (1.01 ~ 1.07)	**0.017**
Q1	1.00 (Reference)		1.00 (Reference)		1.00 (Reference)	
Q2	1.14 (0.85 ~ 1.52)	0.382	1.09 (0.81 ~ 1.46)	0.577	1.03 (0.82 ~ 1.29)	0.789
Q3	1.63 (1.25 ~ 2.14)	**<0.001**	1.51 (1.15 ~ 1.99)	**0.003**	1.72 (1.41 ~ 2.11)	**<0.001**
Q4	2.08 (1.60 ~ 2.71)	**<0.001**	1.74 (1.30 ~ 2.33)	**<0.001**	1.68 (1.39 ~ 2.03)	**<0.001**
ICU mortality at 60-day						
Neutrophil counts/PNI as continuous	1.06 (1.03 ~ 1.08)	**<0.001**	1.03 (0.99 ~ 1.08)	0.123	1.02 (0.99- 1.06)	0.188
Q1	1.00 (Reference)		1.00 (Reference)		1.00 (Reference)	
Q2	1.13 (0.80 ~ 1.59)	0.501	1.09 (0.77 ~ 1.54)	0.633	1.01 (0.77 ~ 1.32)	0.948
Q3	1.62 (1.17 ~ 2.23)	**0.003**	1.52 (1.10 ~ 2.11)	**0.012**	1.33 (1.05 ~ 1.69)	**0.019**
Q4	1.95 (1.43 ~ 2.67)	**<0.001**	1.68 (1.18 ~ 2.37)	**0.004**	1.25 (1.00 ~ 1.57)	0.055
In-hospital mortality at 90-day						
Neutrophil counts/PNI as continuous	1.06 (1.04 ~ 1.09)	**<0.001**	1.04 (1.01 ~ 1.07)	**0.035**	1.04 (1.01 ~ 1.07)	**0.017**
Q1	1.00 (Reference)		1.00 (Reference)		1.00 (Reference)	
Q2	1.14 (0.85 ~ 1.52)	0.382	1.09 (0.81 ~ 1.46)	0.577	1.03 (0.82 ~ 1.29)	0.789
Q3	1.63 (1.25 ~ 2.14)	**<0.001**	1.51 (1.15 ~ 1.99)	**0.003**	1.72 (1.41 ~ 2.11)	**<0.001**
Q4	2.08 (1.60 ~ 2.71)	**<0.001**	1.74 (1.30 ~ 2.33)	**<0.001**	1.68 (1.39 ~ 2.03)	**<0.001**
ICU mortality at 90-day						
Neutrophil counts/PNI as continuous	1.06 (1.03 ~ 1.08)	**<0.001**	1.03 (0.99 ~ 1.08)	0.123	1.02 (0.99- 1.06)	0.188
Q1	1.00 (Reference)		1.00 (Reference)		1.00 (Reference)	
Q2	1.13 (0.80 ~ 1.59)	0.501	1.09 (0.77 ~ 1.54)	0.633	1.01 (0.77 ~ 1.32)	0.948
Q3	1.62 (1.17 ~ 2.23)	**0.003**	1.52 (1.10 ~ 2.11)	**0.012**	1.33 (1.05 ~ 1.69)	**0.019**
Q4	1.95 (1.43 ~ 2.67)	**<0.001**	1.68 (1.18 ~ 2.37)	**0.004**	1.25 (1.00 ~ 1.57)	0.055

* PNI: Prognostic Nutritional Index; Q1 (Quartile 1; Neutrophil counts/PNI ≤ 0.72, n=529), Q2 (Quartile 2; 0.72 < Neutrophil counts/PNI ≤ 1.28, n=529), Q3 (Quartile 3; 1.28 < Neutrophil counts/PNI ≤ 2.10, n=529) and Q4 (Quartile 4; Neutrophil counts/PNI > 2.10, n=529). HR, hazard ratio; CI, confidential interval.

Model 1: Cox univariate analysis.

Model 2: Adjusted for age, gender, height, weight, race, languages, insurance and marital status.

Model 3: Adjusted for age, gender, height, weight, race, languages, insurance and marital status, WBC, RBC, platelet, hemoglobin, chloride, continuous renal replacement therapy, mechanical ventilation, hypertension, type 2 diabetes mellitus, heart failure, myocardial infarction, malignant tumor, chronic kidney disease, acute renal failure, cirrhosis, hepatitis, pneumonia, stroke, hyperlipemia, acute kidney injury and chronic obstructive pulmonary disease, SOFA score, APSIII score, SAPSII score, OASIS score, Charlson score.

Bold red font indicates P<0.05, indicating statistical significance.

These findings consistently illustrate a significant association between higher Neutrophil counts/PNI quartiles and increased short- to medium-term mortality rates in critically ill sepsis patients.

### 3.4 RCS regression models for all-cause mortality (in hospital and ICU)

**Figure 3:**
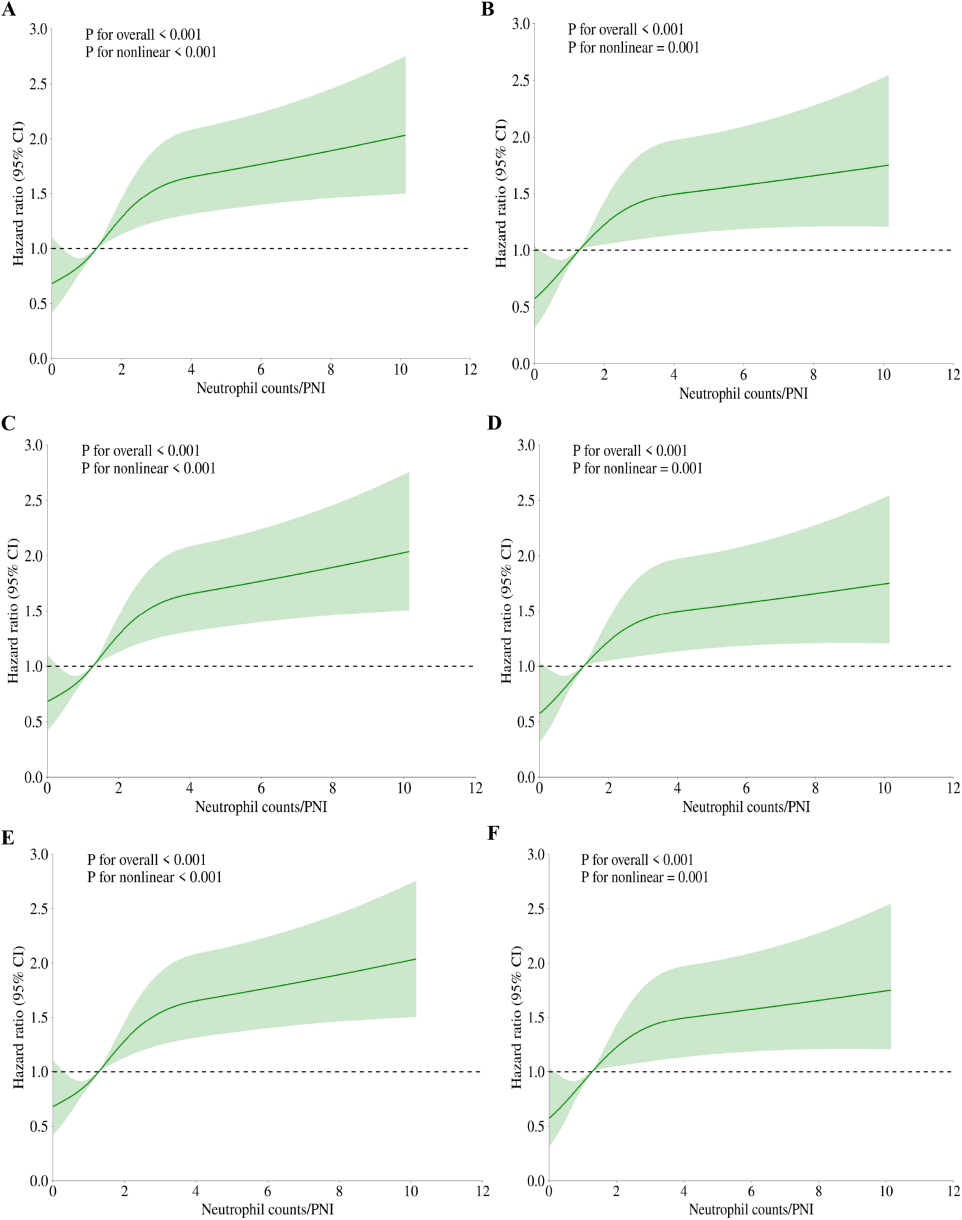
RCS regression for Neutrophil counts/PNI and mortality. **(A)** RCS for 30-day hospital mortality in univariate analysis. **(B)** RCS for 30-day ICU mortality in univariate analysis. **(C)** RCS for 60-day hospital mortality in univariate analysis. **(D)** RCS for 60-day ICU mortality in univariate analysis. **(E)** RCS for 90-day hospital mortality in univariate analysis. **(F)** RCS for 90-day ICU mortality in univariate analysis. The p-values shown in the figures were calculated by the likelihood ratio test of the spline model against the null model. All p-values for nonlinearity were below 0.001.

We utilized the restricted cubic spline (RCS) regression model to further investigate the risk, uncovering a nonlinear association between Neutrophil counts/PNI and mortality. **Figure 3** depict the outcomes of both univariate and multivariate analyses that examine the relationship between Neutrophil counts/PNI and mortality in both hospital and ICU settings. For in-hospital mortality, the overall effect P-value was <0.001 at 30-day (**Figure 3A**), 60-day (**Figure 3C**), and 90-day (**Figure 3E**) intervals, with the P-value for the nonlinear effect also <0.001 before adjustment, indicating a significant association. Similarly, regarding ICU mortality, the P-value for the overall effect was <0.001 at 30 days (**Figure 3B**), 60 days (**Figure 3D**), and 90 days (**Figure 3F**), with the P-value for the nonlinear effect being <0.001 prior to adjustment.

**Figure 4:**
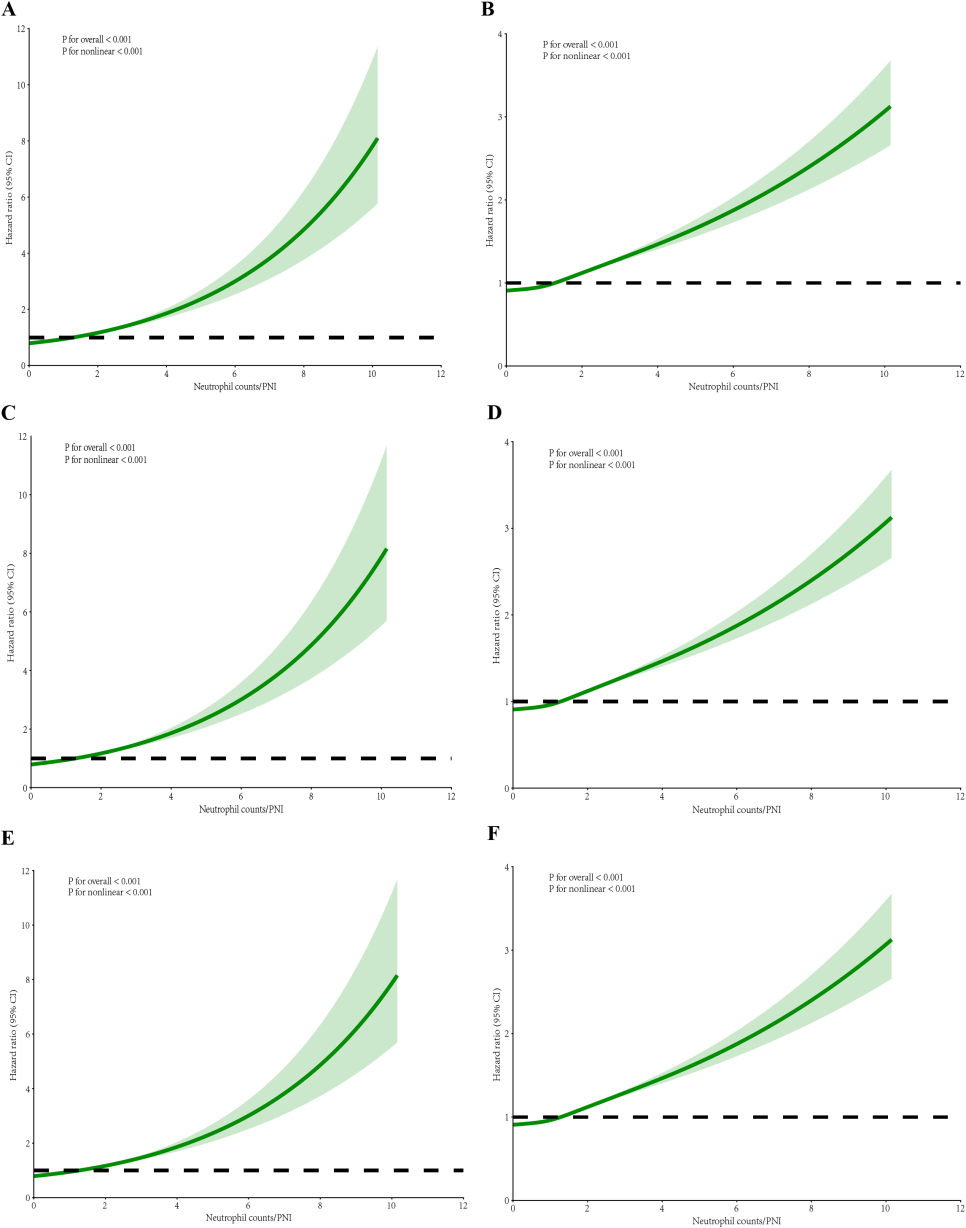
RCS regression for Neutrophil counts/PNI and mortality after adjustment. **(A)** RCS for 30-day hospital mortality in multivariate analysis. **(B)** RCS for 30-day ICU mortality in multivariate analysis. **(C)** RCS for 60-day hospital mortality in multivariate analysis. **(D)** RCS for 60-day ICU mortality in multivariate analysis. **(E)** RCS for 90-day hospital mortality in multivariate analysis. **(F)** RCS for 90-day ICU mortality in multivariate analysis. The p-values shown in the figures were calculated by the likelihood ratio test of the spline model against the null model. All p-values for nonlinearity were below 0.001. Moreover, the images showed a J-shaped association between the Neutrophil counts/PNI and mortality in ICU patients with sepsis.

After adjustment, all P-values remained below 0.001, further substantiating the nonlinear relationship. The visualization of these analyses illustrates a J-shaped curve between Neutrophil counts/PNI and mortality among ICU patients with sepsis. These findings consistently highlight a nonlinear relationship between Neutrophil counts/PNI and patient mortality across both settings. As demonstrated in **Figures 3**, **4**, the inflection point in both multifactorial models occurs at a Neutrophil counts/PNI value of 1.7.

### 3.5 Subgroup analysis

**Figure 5:**
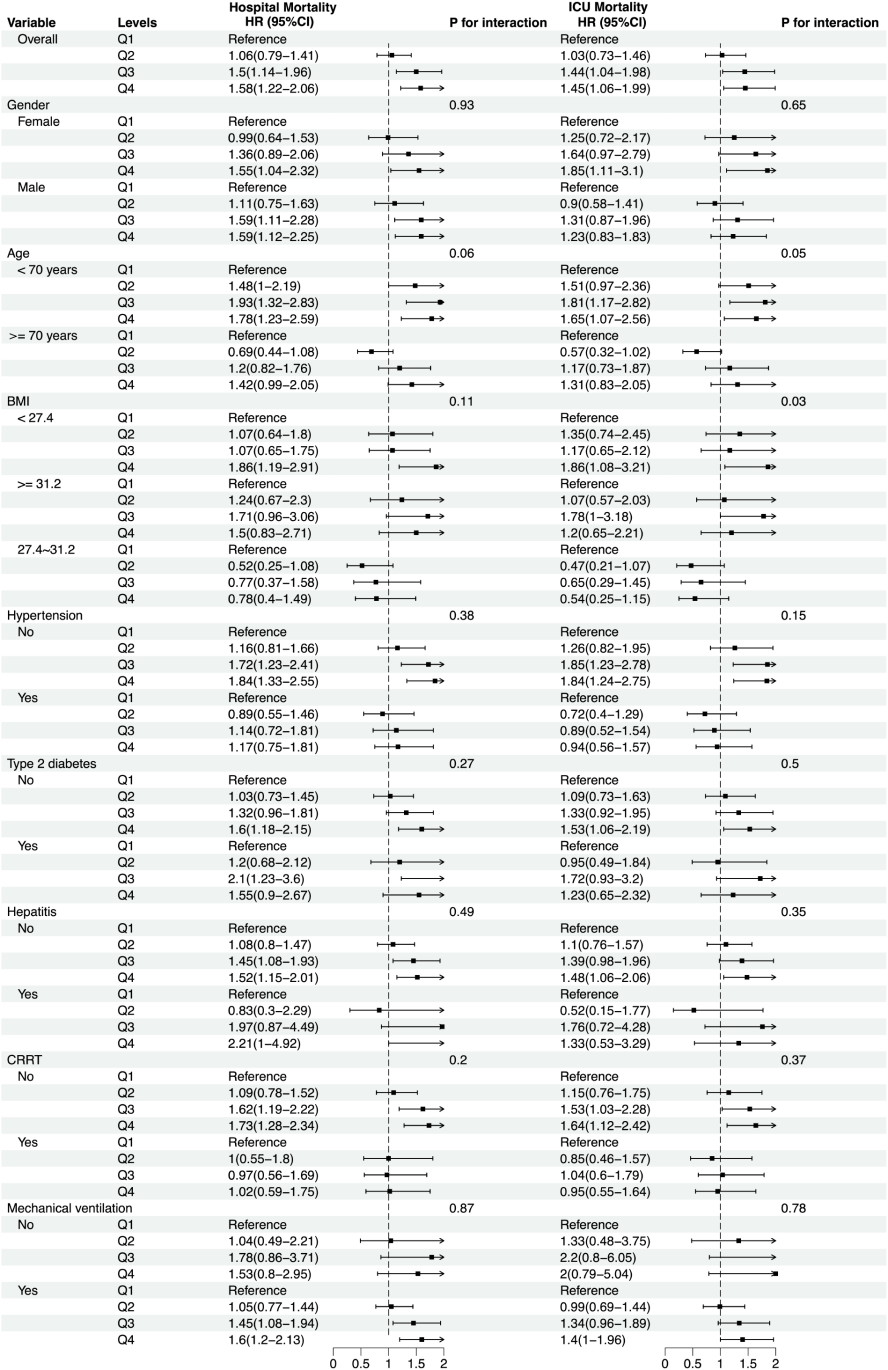
Forest plots for different subgroup analyses of HRs for the association between Neutrophil counts/PNI and in-hospital mortality and ICU mortality. HR, Hazard Risk. Continuous Renal Replacement Therapy.

In the subgroup analysis, the direction of effect estimates within various subgroups was consistent with the overall study outcomes. Analyses were stratified by variables including age (<70 years and ≥70 years), gender, BMI (<27.4 kg/m², 27.4-31.2 kg/m², ≥31.2 kg/m²), and the presence of conditions such as hypertension, type 2 diabetes, hepatitis, as well as the use of mechanical ventilation and continuous renal replacement therapy. As shown in **Figure 5**, no significant interactions were observed between Neutrophil counts/PNI and these factors in relation to in-hospital mortality, with all interaction P-values exceeding 0.05.

However, for ICU mortality, a significant interaction effect was identified with BMI (interaction P = 0.03), suggesting that the impact of Neutrophil counts/PNI on mortality may differ within specific subgroup populations, particularly concerning BMI. These findings underscore the importance of considering individual patient characteristics, such as BMI, when evaluating the prognostic significance of Neutrophil counts/PNI in critically ill sepsis patients.

### 3.6 Neutrophil counts/geriatric nutritional risk index for elderly patients

A total of 929 elderly patients with severe sepsis (aged 70 years or older) were included in the analysis. After grouping according to the quartiles of the Neutrophil counts/Geriatric Nutritional Risk Index, P25 is 2725.089, P50 is 3193.618, and P75 is 3747.333. Survival curve analysis showed that there was no significant association between Neutrophil counts/Geriatric Nutritional Risk Index (NC/GNRI) and in-hospital mortality and ICU mortality in elderly patients with severe sepsis (P all above 0.05).

30-day In-hospital Mortality: When NC/GNRI was treated as a continuous variable, the hazard ratio (HR) was 1.00 (95% CI: 1.00 ~ 1.00) in all three models, with P-values ranging from 0.360 to 0.403, indicating no significant association. When categorized into quartiles (Q1-Q4), in Model 3 (adjusted for multiple variables including age, gender, and various clinical factors), Q2 showed a statistically significant HR of 0.56 (95% CI: 0.38 ~ 0.82, P=0.003), Q3 showed a significant HR of 0.51 (95% CI: 0.34 ~ 0.76, P<0.001), and Q4 showed a significant HR of 0.66 (95% CI: 0.46 ~ 0.95, P=0.027). This suggests that compared to the reference group (Q1), patients in Q2, Q3, and Q4 had lower risks of 30-day in-hospital mortality.

60-day and 90-day In-hospital Mortality: Similar patterns were observed for 60-day and 90-day in-hospital mortality. In Model 3, for 60-day mortality, Q2 had an HR of 1.08 (95% CI: 0.79 ~ 1.47, P=0.630), Q3 had an HR of 0.82 (95% CI: 0.59 ~ 1.13, P=0.233), and Q4 had an HR of 1.18 (95% CI: 0.87 ~ 1.60, P=0.285). For 90-day mortality, the results were largely insignificant across all quartiles in Model 3.

30-day ICU Mortality: For NC/GNRI as a continuous variable, the HR was 1.00 (95% CI: 1.00 ~ 1.00) across all models, with P-values ranging from 0.174 to 0.403. When categorized, in Model 3, Q2 had an HR of 1.01 (95% CI: 0.77 ~ 1.32, P=0.950), Q3 had an HR of 1.33 (95% CI: 1.05 ~ 1.68, P=0.019), and Q4 had an HR of 1.25 (95% CI: 1.00 ~ 1.57, P=0.055), indicating no statistically significant association for Q2 and marginal significance for Q3 and Q4.

60-day and 90-day ICU Mortality: The results for 60-day and 90-day ICU mortality showed that in Model 3, Q2 had an HR of 0.56 (95% CI: 0.38 ~ 0.82, P=0.003) for 60-day mortality and Q3 had an HR of 0.51 (95% CI: 0.34 ~ 0.76, P<0.001), while Q4 had an HR of 0.66 (95% CI: 0.46 ~ 0.95, P=0.027) for 90-day mortality. These findings indicate a lower risk of ICU mortality in certain quartiles compared to the reference group. (**Supplementary Table 1**)

## 4 Discussion

### 4.1 Main findings and significance

This study introduces a novel prognostic biomarker, the Neutrophil counts/Prognostic Nutritional Index ratio, designed to forecast outcomes in critically ill patients with sepsis. Our retrospective analysis leverages the extensive data from the MIMIC-IV database, highlighting that higher Neutrophil counts/PNI ratios correlate with increased mortality at various short- to medium-term intervals. These findings suggest a significant potential for this biomarker to enhance risk stratification and inform clinical decision-making in intensive care settings. Our research overcomes the individual limitations of neutrophil counts and PNI by integrating them, thus addressing both the inflammatory state and the nutritional-immune status of patients in the prognostic evaluation process. This dual approach represents a significant shift in prognostic evaluations, potentially improving patient management through more tailored treatment strategies.

### 4.2 Neutrophil counts/PNI ratio and other prognostic biomarkers of sepsis

In clinical practice, neutrophil counts serve as a fundamental marker for evaluating the acute inflammatory response. These counts are prominently elevated in the setting of infectious diseases, acting as a crucial component in emergency and critical care assessments to distinguish between bacterial infections and other etiologies (Zhu et al., 2024). Within oncology, elevated neutrophil levels have been linked with poor prognosis, as they reflect the systemic inflammatory milieu often associated with tumor growth and metastasis (Timamy et al., 2024). Despite their widespread use as an indicator of immune response, neutrophil counts are easily influenced by confounding factors such as concurrent infections, inflammation from non-septic sources, medications, or physiological stressors, which can obscure their specificity in determining prognosis in sepsis (Feng et al., 2024; Lee et al., 2025). The neutrophil-to-PNI ratio emerged as a superior prognostic tool compared to isolated biomarkers such as neutrophil count, absolute lymphocyte count, or albumin, addressing a critical gap in sepsis risk stratification. While neutrophil count reflects acute neutrophilic hyperinflammation and PNI captures chronic immunonutritional compromise (lymphopenia and hypoalbuminemia), their integration into a single ratio provides a dynamic snapshot of the dual-hit pathophysiology in sepsis—simultaneous hyperinflammation and immunosuppression (Zhu et al., 2024; Lee et al., 2025). For instance, a patient with elevated neutrophil count but normal PNI (≥38) may exhibit transient infection-driven inflammation, whereas the same neutrophil count combined with a low PNI (<35) signals compounded risks of malnutrition and immune exhaustion, portending worse outcomes (Feng et al., 2024). The recent evidence advocating composite indices to overcome the “one-dimensional” limitations of standalone markers in heterogeneous sepsis populations (Sun et al., 2020). Clinically, the ratio’s J-shaped association with mortality (**Figure 4**) identifies actionable thresholds: patients with ratios >1.7 faced a 2.1-fold mortality risk (HR: 2.08, 95% CI: 1.60–2.71), suggesting intensified monitoring or immunonutritional support for this subgroup. Importantly, the ratio’s calculation is both logistically feasible and cost-effective, requiring only routine laboratory parameters. In resource-limited settings, where advanced biomarkers (e.g., procalcitonin trends or cytokine panels) may be unavailable, this ratio offers a pragmatic alternative. Future protocols could automate its calculation within electronic health records, triggering real-time alerts for high-risk patients—a strategy proven effective in sepsis bundles (Saxena et al., 2024). Nevertheless, prospective validation is warranted to confirm its utility in guiding targeted therapies (e.g., granulocyte colony-stimulating factor in neutropenic sepsis or albumin supplementation in hypoalbuminemia).

Conversely, the PNI offers valuable insights into a patient’s nutritional and immune status, prominently utilized in chronic illness contexts, such as cancer, to anticipate treatment responses and potential outcomes. As a measure derived from serum albumin and lymphocyte counts, the PNI aids in gauging chronic inflammation levels, nutritional deficiencies, and overall immune competence. A multicenter study (Takahashi et al., 2025) revealed that immunonutritional assessment using PNI would provide useful prognostic information for patients with resectable non-small cell lung cancer. Bermudez-Pineda et al (Bermúdez-Pineda et al., 2025). used it to predict clinical outcomes in gynecological cancer. Solano et al (Solano et al., 2025). used PNI to evaluate the clinical characteristics and prognosis of patients with heart failure, and more researchers combined PNI with other biomarkers to predict the prognosis of diseases, such as miR-132-3p in the prediction of gastric cancer (Qin et al., 2025). Or combined with D-dimer levels to predict the prognosis of cancer after gastrectomy (Yamamoto et al., 2025). Many meta-analyses (Xu et al., 2023; Chen et al., 2024; Zhang and Su, 2024) have also reviewed and summarized the efficacy of PNI in predicting the outcome of patients with different pathological states, and some scholars have found that there is a certain correlation between preoperative prognostic nutritional index and the risk of postoperative delirium (Hung et al., 2023). However, its role diminishes in acute care settings where rapid physiological changes necessitate indicators that reflect immediate health statuses. Combining these seemingly disparate metrics into the neutrophil-to-PNI ratio represents an innovative synthesis designed specifically to tackle their individual limitations. By bridging the acute inflammatory response with a comprehensive view of the patient’s nutritional and immune baseline, our approach presents a holistic snapshot of the patient’s condition. Notably, neutrophil counts alone fail to account for the nutritional and immunologic alterations that patients undergo, while the PNI’s long-term perspective neglects the acute shifts critically ill patients experience. This advantageously positions the Neutrophil counts/PNI/PNI ratio over existing standalone biomarkers, as it is capable of providing more nuanced interpretations of patient conditions particularly relevant during the dynamic physiological shifts characteristic of sepsis. However, it is important to acknowledge the heterogeneity of sepsis patients, which may influence the ratio’s predictive performance. Sepsis manifests in various subtypes, each with distinct pathophysiologies and clinical presentations. For instance, some subtypes may be driven predominantly by bacterial infections, while others may be secondary to viral or fungal pathogens (Yang et al., 2024). These differences can affect the inflammatory and immune responses, potentially altering the relationship between the Neutrophil counts/PNI ratio and mortality. Future research should explore how these subtypes, along with other factors such as age, comorbidities, and genetic predispositions, interact with the ratio to produce different results. This will be crucial for refining the ratio’s application in specific patient populations and enhancing its clinical utility. Beyond individual patient outcomes, the Neutrophil counts/PNI ratio can potentially facilitate a more unified and standardized approach to sepsis assessment across different healthcare institutions.

This consistency can prove invaluable for multicentric research, clinical trials, and the development of sepsis management guidelines, ensuring that practices are universally applicable despite local variability in disease presentation. Additionally, under global health policies aiming for resource optimization, the Neutrophil counts/PNI ratio could guide treatment prioritization, identifying patients most in need of immediate intervention versus those who may benefit from more conservative management approaches. Thus, the integrated ratio provides a balanced view, encapsulating the immediate inflammatory surge while recognizing the foundational nutritional immunological reserve. This presents a more precise stratification, reducing misclassifications that might arise when relying solely on neutrophil counts or nutritional indices.

### 4.3 Potential association between body response and neutrophil counts/PNI in sepsis

Sepsis represents a notorious challenge in critical care, marked by complex interactions between the infectious agents and host responses, leading to exacerbated inflammatory paths and severe metabolic disturbances. The initial phase of sepsis is dominated by intense inflammation, spearheaded by neutrophil activation (Xue et al., 2024), which is crucial to the innate immune response. Neutrophils, responsive to cytokine signaling, migrate to infection sites and play an integral role in pathogen clearance through phagocytosis and the release of antimicrobial proteins (Moura and Siqueira, 2025). However, excessive or prolonged activation contributes to tissue injury, perpetuating a cycle of ineffective inflammation (Nakama et al., 2024) that results in further systemic damage and progression to severe sepsis.

Meanwhile, adaptive immune responses are visibly altered in sepsis, evidenced by lymphopenia (Wang et al., 2024) — an indicator captured duly within the PNI. Lymphocyte apoptosis, attributed to elevated Fas ligand expressions (Medica et al., 2024) and persistent tissue necrosis factor levels (Cakmak et al., 2025), leads to substantial reductions in lymphocyte counts. This compromises the host’s ability to mount effective adaptive immune responses, leaving patients vulnerable to opportunistic infections and contributing to poorer prognoses. In parallel, the inflammatory milieu exerts significant influence over metabolic pathways. Sepsis-associated hypoalbuminemia results largely from systemic capillary leak syndrome and hepatic reprioritization of protein synthesis towards acute-phase reactants over albumin (Cakmak et al., 2025). Hypoalbuminemia is a consistent prognostic marker for morbidity in sepsis and reflects both inflammation and malnourishment.

These metabolic and immune challenges further underscore the rationale for utilizing the Neutrophil counts/PNI ratio, which adeptly encapsulates these tumultuous physiological changes. The selected biomarkers here—neutrophils, lymphocytes, and albumin—represent critical nodes within the inflammatory-nutritional network intricately involved in sepsis pathophysiology. Neutrophils mirror acute responses, lymphocytes underscore longer-term adaptive capabilities, and serum albumin highlights nutritional sufficiency, forming a composite tool that traverses the acute-chronic spectrum of sepsis impacts. Our choice of these parameters also facilitates timely clinical responses to the developing stages of sepsis. For instance, a rising Neutrophil counts/PNI ratio acts as a harbinger of impaired patient prognosis, thereby triggering preemptive strategies to mitigate inflammation and bolster nutritional support (Islam et al., 2024), ultimately aiming to recalibrate the immune system’s balance and restore homeostasis. Such a proactive approach, guided by fluid biomarker interpretation, aligns with the current trends towards precision medicine in critical care environments.

### 4.4 Clinical and prognostic implications of the NC/PNI in sepsis management

Our research carries vital implications for sepsis management and could revolutionize prognostic evaluation, thereby enhancing the quality of patient treatment regimes. By consistently incorporating the Neutrophil counts/PNI ratio into ICU assessments, clinicians can craft more patient-focused care plans. These plans account for individual differences in inflammatory response and nutritional status, which may greatly boost the specificity and sensitivity of sepsis diagnosis. This enables a swift, evidence-based approach to interventions, such as enhanced nutritional support, refined anti-inflammatory therapies, or adjusted antimicrobial tactics (Hongya et al., 2024).

From a healthcare system perspective, using the Neutrophil counts/PNI ratio could reduce mortality rates and improve cost-efficiency in sepsis management. This might lead to shorter hospital stays, less need for invasive procedures, and better ICU resource utilization, all of which bring clear economic benefits. Furthermore, the predictive precision of the Neutrophil counts/PNI ratio could help develop treatment algorithms that prevent sepsis complications and reduce post-intervention morbidity (Baek et al., 2024). These medical and organizational improvements can enhance public health by boosting productivity and reducing absenteeism due to better patient recovery outcomes.

The calculation of the Neutrophil counts/PNI ratio is both feasible and cost-effective as it relies on standard critical care measurements: neutrophil counts, serum albumin, and lymphocyte counts. This is especially beneficial in resource-limited settings where advanced diagnostic tools may not be readily available. By combining these parameters, the ratio offers a dynamic view of a patient’s inflammatory and nutritional-immune status, providing a more complete assessment for clinicians. Our study shows a J-shaped link between the Neutrophil counts/PNI ratio and mortality, indicating critical thresholds for clinical decisions. For example, patients with a ratio above 1.7 have a significantly higher mortality risk. This suggests the need for closer monitoring and targeted interventions like nutritional support or immunomodulatory therapies, in line with precision medicine principles. Such an approach could improve patient outcomes through timely, tailored interventions.

However, to translate this innovation into clinical practice, further validation is needed. Prospective studies across diverse populations are essential to confirm the reliability and generalizability of the Neutrophil counts/PNI ratio. Clear thresholds for risk stratification and integration of the ratio into clinical algorithms are also crucial for its wider adoption. Future research should explore how this biomarker can guide specific treatments, such as adjusting nutritional support intensity or informing antimicrobial stewardship decisions. While our study establishes a significant association between an elevated neutrophil-to-PNI ratio and increased mortality in sepsis, establishing a causal relationship is paramount for translating this finding into actionable clinical interventions. Observational associations, such as ours, are susceptible to confounding by unmeasured or incompletely adjusted factors (e.g., underlying disease severity trajectories, specific microbial pathogens, or concurrent treatments not captured in our data), inherently limiting definitive causal inference regarding the ratio’s direct biological impact. Future research should therefore adopt the rigorous framework of target trial emulation (Hernán et al., 2022) to address this causality gap. This involves designing a hypothetical randomized trial (e.g., ‘Does intervening to lower a high neutrophil-to-PNI ratio improve survival?’) and emulating it using observational data through key steps: defining explicit eligibility criteria; specifying clear treatment strategies based on the ratio (e.g., ‘standard care plus ratio-lowering therapy’ vs. ‘standard care alone’ at defined thresholds); using propensity score matching or weighting (IPTW) to create comparable groups; defining protocolized outcomes (e.g., 28-day mortality) with precise time-zero; and crucially, employing marginal structural models (MSMs) with IPTW or g-methods to handle time-varying confounding. If such analyses robustly demonstrate a causal beneficial effect of lowering the ratio, it would provide a strong rationale for developing and testing targeted interventions (e.g., specific immunomodulatory or nutritional strategies) in high-risk patients identified by this biomarker, potentially transforming it from a prognostic indicator into a theragnostic tool for personalized sepsis management.

In our study, we also delved into the prognostic utility of the NC/GNRI for elderly patients with severe sepsis. We found no significant link between NC/GNRI and in-hospital/ICU mortality via survival curve analysis (P>0.05). As a continuous variable, NC/GNRI showed no significant association with 30-day in-hospital mortality (HR=1.00, 95% CI:1.00~1.00, P=0.360-0.403). However, when divided into quartiles, Q2, Q3, and Q4 exhibited lower risks than Q1 in Model 3 (HR=0.56, P=0.003; HR=0.51, P<0.001; HR=0.66, P=0.027). Similar trends were seen in 60-day and 90-day in-hospital mortality, but with largely insignificant results. For ICU mortality, the results were less consistent, showing no significant association for Q2 and marginal significance for Q3 and Q4 in 30-day analysis. Yet, certain quartiles indicated lower mortality risks in 60-day and 90-day ICU mortality analyses. The NC/GNRI is a specialized indicator designed for the elderly population, taking into account the physiological characteristics and nutritional needs of older adults. Unlike the general PNI, the GNRI places greater emphasis on the patient’s weight and protein nutritional status, making it suitable for assessing the nutritional risks of older adults, including those with critical conditions. The results of our study suggest that the NC/GNRI may have a complex relationship with mortality in elderly patients with severe sepsis. The significant findings for 30-day in-hospital mortality indicate that elderly patients in higher quartiles of NC/GNRI may have a lower risk of mortality. This could be attributed to the fact that higher NC/GNRI values reflect a better nutritional status and stronger immune function in these patients, which may enhance their ability to combat severe sepsis and its complications. However, the lack of significant associations in the 60-day and 90-day in-hospital mortality and the inconsistent results for ICU mortality suggest that the prognostic value of NC/GNRI may be more pronounced in the short term. This may be due to the dynamic nature of the nutritional and immune status of elderly patients during the course of severe sepsis, where initial nutritional status may have a greater impact on short-term outcomes, while other factors such as treatment response and disease progression may play a more significant role in longer-term outcomes. The results of this study provide valuable insights for the clinical management of elderly patients with severe sepsis. They highlight the importance of considering both the inflammatory response and nutritional status in the prognostic evaluation of these patients. While the NC/GNRI shows potential as a prognostic tool for short-term mortality in elderly patients with severe sepsis, further research is needed to validate its utility in different elderly populations and to explore its potential role in guiding nutritional support and other therapeutic interventions. Additionally, the findings underscore the need for a comprehensive approach to the care of elderly patients with severe sepsis, incorporating assessments of both nutritional status and inflammatory response to optimize patient outcomes.

### 4.5 Limitations

While the findings of this study present significant advances in sepsis prognosis and management, there are several limitations that are important to acknowledge. These stem from the retrospective nature of our research design, the characteristics of the MIMIC database, the specific data utilized, the formulation of the biomarker itself, and the statistical methods employed (Diaz Ochoa et al., 2024). The retrospective nature of our study inherently introduces certain limitations. Such studies are prone to selection bias as the dataset comprises previously collected data, which might not fully represent the broader population of interest (Casaer et al., 2011). There is also the risk of information bias, given that data quality is contingent upon the accuracy of the original recording. Retrospective design does not allow for control over data collection variables, leading to challenges in delineating causal relationships and often restricting findings to associations. This could potentially affect the robustness and applicability of the conclusions drawn in a real-world clinical setting. In addition, although the MIMIC-IV database is comprehensive and extensively utilized in critical care research, it predominantly represents patients from a single geographic region, specifically in the northeastern United States. This poses significant limitations in terms of diversity and generalizability, as the dataset might not adequately reflect global populations or even diverse ethnic groups within the United States. Additionally, within the MIMIC database, a significant proportion of the population is white, introducing ethnic homogeneity that may not accurately portray the varied genotypic and phenotypic responses to sepsis present in a more diverse patient cohort. Our findings, derived from the MIMIC-IV database, require external validation in independent cohorts. Although our medical center has also collected some clinical indicator information of relevant patients, there is a significant gap in the comprehensiveness and completeness of the information compared to the MIMIC database. Many key indicators have serious data missing, making it difficult to meet the data standards required for external validation. Future studies should validate the neutrophil-to-PNI ratio in geographically diverse settings (e.g., Asian, European cohorts) and prospective registries to confirm its universal prognostic utility. This lack of diversity limits the ability to generalize study findings to broader, heterogeneous populations, where genetic, environmental, and cultural factors might influence disease presentation and progression. Incomplete data is another critical limitation faced in this study. During data extraction and cleaning processes, several predictors with more than 30% missing data were excluded from analysis. This constriction may introduce biases if the missing data correlates with specific unmeasured factors affecting outcomes. Such exclusions and limitations in data integrity might lead to an underestimation or overestimation of the Neutrophil counts/PNI ratio’s predictive capability. Moreover, the cohort used in this analysis comprised patients primarily undergoing first-time ICU admission and could exclude individuals with recurrent issues or comorbidities, which could skew the relevance of the findings to more complex or atypical patient conditions.

Due to constraints in the MIMIC-IV database, we were unable to account for confounding conditions that independently alter neutrophil counts, such as corticosteroid therapy, acute stress responses, smoking status, active malignancies, or chronic inflammatory diseases. For instance, corticosteroids are known to induce neutrophilia by delaying apoptosis and promoting demargination, which could artificially inflate the neutrophil-to-PNI ratio and overestimate sepsis-related inflammation in treated patients (Cox, 1995). Similarly, smokers often exhibit baseline neutrophilia due to chronic airway inflammation, potentially distorting risk stratification (Arnson et al., 2010). These unmeasured confounders may introduce misclassification bias, particularly in patients where elevated neutrophil counts reflect non-septic etiologies. Second, while our models adjusted for comorbidities, granular data on disease activity or treatment histories were unavailable, limiting our ability to disentangle sepsis-specific effects from chronic inflammatory states. Future studies incorporating detailed medication records, smoking histories, and biomarkers of stress (e.g., cortisol) are needed to validate the ratio’s robustness across heterogeneous populations.

Our study is also limited by the skewed distribution of PNI values in the cohort, with 99.3% of patients classified as having severe malnutrition (PNI<35) and no individuals in the moderate malnutrition range (35≤PNI<38). This precluded stratification by PNI severity categories, as suggested by the reviewer, and highlights the challenges of applying generalized nutritional indices to critically ill sepsis populations, where profound hypoalbuminemia and lymphopenia are nearly universal (Pai Mangalore et al., 2022). While this limits direct comparisons with prior studies using PNI categories, our focus on the neutrophil-to-PNI ratio as a continuous variable circumvented arbitrary thresholds and revealed its J-shaped association with mortality. Future research should validate this ratio in mixed cohorts (e.g., non-ICU sepsis patients) with more balanced nutritional profiles to confirm its generalizability.

The biomarkers utilized in the creation of the Neutrophil counts/PNI ratio bring their own set of limitations. Neutrophil counts can vary widely due to factors unrelated to sepsis, including physiological stress, medications, and other non-septic inflammatory conditions (Hernandez-Beeftink et al., 2022; Schneider et al., 2025), potentially leading to confounding within this ratio. Similarly, serum albumin levels can be influenced not only by inflammation and nutritional status but also by factors such as fluid balance and liver function abnormalities (Sun et al., 2020), making it a less specific indicator of sepsis-related nutritional compromise. Formulating a composite biomarker using these parameters may lead to dilution effects, where extreme abnormalities in one component could overshadow the relevant insights provided by the others, thus affecting the ratio’s practical utility.

Then, the statistical methods employed, including Cox proportional hazards models and Kaplan-Meier survival curves, while robust in many respects, come with constraints that impact the interpretation of results. The Cox model assumes proportional hazards, which might not necessarily hold true across all patient subgroups, potentially confounding findings. Though adjustments were made for a wide range of confounding variables, residual confounding remains a possibility given the inherent complexities of sepsis. The use of restricted cubic splines to examine nonlinear relationships, while flexible, depends heavily on the chosen reference points, which can sometimes skew interpretation of the association curves if not carefully managed (Wu and Huang, 2024).

Another key limitation is the potential for collinearity or overfitting due to the inclusion of multiple overlapping severity scores (SOFA, APSIII, SAPSII, OASIS). While these scores were included to provide a comprehensive assessment of patient severity and validate the consistency of the Neutrophil counts/PNI ratio’s prognostic value across different metrics, their combined inclusion may introduce redundancy. We did not perform formal collinearity diagnostics, such as variance inflation factor (VIF) analysis, as our primary focus was on the independent contribution of the Neutrophil counts/PNI ratio rather than on the interrelationships among the severity scores. Given the large sample size and the clinical relevance of these scores, we prioritized a comprehensive adjustment for patient severity over concerns about collinearity. However, this approach may limit the interpretability of the individual effects of each severity score. Despite these limitations, we believe our study provides valuable insights into the potential of the Neutrophil counts/PNI ratio as a prognostic tool for septic patients. By validating our indicator through a systematic examination of MIMIC data, we aim to deliver insights that could redefine prognostic evaluations for septic patients and enhance clinical decision-making processes.

Lastly, the subgroup analyses, though comprehensive, were not powered to detect differences within all stratifications, especially among smaller subgroups. This limitation necessitates caution in interpreting these analyses and underscores the need for further validation studies tailored to diverse population bases, which could more accurately elucidate logistic concerns and verify the practical utility of the Neutrophil counts/PNI ratio in varied clinical scenarios. While these limitations temper our findings, they most importantly serve as essential considerations for the design of future research.

## 5 Conclusions

In conclusion, our study establishes the Neutrophil counts/PNI ratio as a novel and comprehensive biomarker for evaluating sepsis outcomes in ICU settings. This dual-parameter approach offers improved predictive accuracy by integrating immune status and nutritional health, potentially transforming sepsis management practices. While existing limitations underpin this retrospective analysis, the promising outcomes advocate for further prospective studies to validate and incorporate the Neutrophil counts/PNI ratio in routine clinical evaluations, heralding a significant advancement for critical care practitioners.

## Data Availability

The datasets presented in this study can be found in online repositories. The names of the repository/repositories and accession number(s) can be found in the article/**Supplementary Material**.
