# Supplementary material for: The J-shaped association between the ratio of neutrophil counts to prognostic nutritional index and mortality in ICU patients with sepsis: a retrospective study based on the MIMIC database
Source: Front Cell Infect Microbiol. 2025 Jul 22;15:1603104. doi: 10.3389/fcimb.2025.1603104 (PMC12322749; doi:10.3389/fcimb.2025.1603104)
Supplement: Supplementary file 1 [file DataSheet1.docx]

| Supplementary Table 1. The association between Neutrophil counts/Geriatric Nutritional Risk Index (NC/GNRI) and in-hospital and ICU mortality in elderly patients (≥70 years) | | | | | | |
| --- | --- | --- | --- | --- | --- | --- |
| Exposure | Model 1 | | Model 2 | | Model 3 | |
|  | HR (95% CI) | *P*-value | HR (95% CI) | *P*-value | HR (95% CI) | *P*-value |
| ***In-hospital mortality at 30-day*** |  |  |  |  |  |  |
| NC/GNRI as continuous | 1.00 (1.00 ~ 1.00) | 0.403 | 1.00 (1.00 ~ 1.00) | 0.360 | 1.00 (1.00 ~ 1.00) | 0.403 |
| Q1 | 1.00 (Reference) |  | 1.00 (Reference) |  | 1.00 (Reference) |  |
| Q2 | 0.14 (0.53 ~ 1.09) | 0.381 | 1.04 (0.76 ~ 1.41) | 0.826 | 0.56 (0.38 ~ 0.82) | **0.003** |
| Q3 | 0.68 (0.47 ~ 0.99) | **0.046** | 0.97 (0.70 ~ 1.34) | 0.845 | 0.51 (0.34 ~ 0.76) | **＜0.001** |
| Q4 | 0.90 (0.63 ~ 1.27) | 0.537 | 1.41 (1.04 ~ 1.92) | **0.026** | 0.66 (0.46 ~ 0.95) | **0.027** |
| ***ICU mortality at 30-day*** |  |  |  |  |  |  |
| NC/GNRI as continuous | 1.00 (1.00 ~ 1.00) | 0.174 | 1.00 (1.00 ~ 1.00) | 0.403 | 1.00 (1.00 ~ 1.00) | 0.174 |
| Q1 | 1.00 (Reference) |  | 1.00 (Reference) |  | 1.00 (Reference) |  |
| Q2 | 0.86 (0.54 ~ 1.37) | 0.533 | 0.55 (0.37 ~ 0.82) | **0.003** | 1.01 (0.77 ~ 1.32) | 0.950 |
| Q3 | 0.74 (0.46 ~ 1.20) | 0.230 | 0.55 (0.37 ~ 0.82) | **0.004** | 1.33 (1.05 ~ 1.68) | **0.019** |
| Q4 | 1.17 (0.76 ~ 1.81) | 0.468 | 0.75 (0.52 ~ 1.08) | 0.117 | 1.25 (1.00 ~ 1.57) | 0.055 |
| ***In-hospital mortality at 60-day*** |  |  |  |  |  |  |
| NC/GNRI as continuous | 1.00 (1.00 ~ 1.00) | 0.396 | 1.00 (1.00 ~ 1.00) | 0.356 | 1.00 (1.00 ~ 1.00) | 0.396 |
| Q1 | 1.00 (Reference) |  | 1.00 (Reference) |  | 1.00 (Reference) |  |
| Q2 | 0.76 (0.52 ~ 1.09) | 0.135 | 1.03 (0.76 ~ 1.41) | 0.839 | 1.08 (0.79 ~ 1.47) | 0.630 |
| Q3 | 0.68 (0.47 ~ 0.99) | **0.045** | 0.96 (0.70 ~ 1.33) | 0.829 | 0.82 (0.59 ~ 1.13) | 0.233 |
| Q4 | 0.89 (0.63 ~ 1.27) | 0.526 | 1.41 (1.04 ~ 1.91) | **0.027** | 1.18 (0.87 ~ 1.60) | 0.285 |
| ***ICU mortality at 60-day*** |  |  |  |  |  |  |
| NC/GNRI as continuous | 1.00 (1.00 ~ 1.00) | 0.174 | 1.00 (1.00 ~ 1.00) | 0.174 | 1.00 (1.00 ~ 1.00) | 0.174 |
| Q1 | 1.00 (Reference) |  | 1.00 (Reference) |  | 1.00 (Reference) |  |
| Q2 | 0.86 (0.54 ~ 1.37) | 0.533 | 0.55 (0.37 ~ 0.82) | **0.003** | 0.56 (0.38 ~ 0.82) | **0.003** |
| Q3 | 0.74 (0.46 ~ 1.20) | 0.230 | 0.55 (0.37 ~ 0.82) | **0.004** | 0.51 (0.34 ~ 0.76) | **＜0.001** |
| Q4 | 1.17 (0.76 ~ 1.81) | 0.468 | 0.75 (0.52 ~ 1.08) | 0.117 | 0.66 (0.46 ~ 0.95) | **0.027** |
| ***In-hospital mortality at 90-day*** |  |  |  |  |  |  |
| NC/GNRI as continuous | 1.00 (1.00 ~ 1.00) | 0.396 | 1.00 (1.00 ~ 1.00) | 0.356 | 1.00 (1.00 ~ 1.00) | 0.396 |
| Q1 | 1.00 (Reference) |  | 1.00 (Reference) |  | 1.00 (Reference) |  |
| Q2 | 0.68 (0.47 ~ 0.99) | 0.134 | 1.03 (0.76 ~ 1.41) | 0.840 | 1.08 (0.79 ~ 1.47) | 0.630 |
| Q3 | 0.89 (0.63 ~ 1.27) | **0.044** | 0.96 (0.70 ~ 1.33) | 0.827 | 0.82 (0.59 ~ 1.13) | 0.233 |
| Q4 | 2.08 (1.60 ~ 2.71) | 0.525 | 1.41 (1.04 ~ 1.91) | **0.028** | 1.18 (0.87 ~ 1.60) | 0.285 |
| ***ICU mortality at 90-day*** |  |  |  |  |  |  |
| NC/GNRI as continuous | 1.00 (1.00 ~ 1.00) | 0.174 | 1.00 (1.00 ~ 1.00) | 0.174 | 1.00 (1.00 ~ 1.00) | 0.174 |
| Q1 | 1.00 (Reference) |  | 1.00 (Reference) |  | 1.00 (Reference) |  |
| Q2 | 0.86 (0.54 ~ 1.37) | 0.533 | 0.55 (0.37 ~ 0.82) | **0.003** | 0.56 (0.38 ~ 0.82) | **0.003** |
| Q3 | 0.74 (0.46 ~ 1.20) | 0.230 | 0.55 (0.37 ~ 0.82) | **0.004** | 0.51 (0.34 ~ 0.76) | **＜0.001** |
| Q4 | 1.17 (0.76 ~ 1.81) | 0.468 | 0.75 (0.52 ~ 1.08) | 0.117 | 0.66 (0.46 ~ 0.95) | **0.027** |
| * NC/GNRI: Neutrophil counts/Geriatric Nutritional Risk Index; P25:2725.089, P50:3193.618, P75:3747.333; HR: hazard ratio; CI: confidential interval.  Model 1: Cox univariate analysis.  Model 2: Adjusted for age, gender, height, weight, race, languages, insurance and marital status.  Model 3: Adjusted for age, gender, height, weight, race, languages, insurance and marital status, WBC, RBC, platelet, hemoglobin, chloride, continuous renal replacement therapy, mechanical ventilation, hypertension, type 2 diabetes mellitus, heart failure, myocardial infarction, malignant tumor, chronic kidney disease, acute renal failure, cirrhosis, hepatitis, pneumonia, stroke, hyperlipemia, acute kidney injury and chronic obstructive pulmonary disease, SOFA score, APSIII score, SAPSII score, OASIS score, Charlson score. | | | | | | |
